# SolCyc: a database hub at the Sol Genomics Network (SGN) for the manual curation of metabolic networks in *Solanum* and *Nicotiana* specific databases

**DOI:** 10.1093/database/bay035

**Published:** 2018-05-10

**Authors:** Hartmut Foerster, Aureliano Bombarely, James N D Battey, Nicolas Sierro, Nikolai V Ivanov, Lukas A Mueller

**Affiliations:** 1 Boyce Thompson Institute, 533 Tower Road, Ithaca, New York, 14853-1801, USA; 2 Department of Horticulture, Virginia Polytechnic Institute and State University, 220 Ag Quad Lane, Blacksburg, VA 24061, USA; 3 PMI R&D, Philip Morris Products S.A (Part of Philip Morris International group of companies), Quai Jeanrenaud 6, Neuchâtel CH-2000, Switzerland

## Abstract

SolCyc is the entry portal to pathway/genome databases (PGDBs) for major species of the *Solanaceae* family hosted at the Sol Genomics Network. Currently, SolCyc comprises six organism-specific PGDBs for tomato, potato, pepper, petunia, tobacco and one Rubiaceae, coffee. The metabolic networks of those PGDBs have been computationally predicted by the pathologic component of the pathway tools software using the manually curated multi-domain database MetaCyc (http://www.metacyc.org/) as reference. SolCyc has been recently extended by taxon-specific databases, i.e. the family-specific SolanaCyc database, containing only curated data pertinent to species of the nightshade family, and NicotianaCyc, a genus-specific database that stores all relevant metabolic data of the *Nicotiana* genus. Through manual curation of the published literature, new metabolic pathways have been created in those databases, which are complemented by the continuously updated, relevant species-specific pathways from MetaCyc. At present, SolanaCyc comprises 199 pathways and 29 superpathways and NicotianaCyc accounts for 72 pathways and 13 superpathways. Curator-maintained, taxon-specific databases such as SolanaCyc and NicotianaCyc are characterized by an enrichment of data specific to these taxa and free of falsely predicted pathways. Both databases have been used to update recently created *Nicotiana*-specific databases for *Nicotiana tabacum*, *Nicotiana benthamiana*, *Nicotiana sylvestris* and *Nicotiana tomentosiformis* by propagating verifiable data into those PGDBs. In addition, in-depth curation of the pathways in *N.tabacum* has been carried out which resulted in the elimination of 156 pathways from the 569 pathways predicted by pathway tools. Together, in-depth curation of the predicted pathway network and the supplementation with curated data from taxon-specific databases has substantially improved the curation status of the species–specific *N.tabacum* PGDB. The implementation of this strategy will significantly advance the curation status of all organism-specific databases in SolCyc resulting in the improvement on database accuracy, data analysis and visualization of biochemical networks in those species.

**Database URL:**

https://solgenomics.net/tools/solcyc/

## Introduction

The post genomic era has seen the rise of new technologies and intensified research focusing on function and regulation of genes and their encoded proteins in the cell and within complex metabolic networks (1). With the arrival of high throughput technologies facilitating proteomic and metabolomics studies, the analysis of large volumes of data and their translation into biological context has become much more feasible and enabled a broader approach toward deciphering metabolic processes (2, 3). The ongoing development of Omics technologies allows tapping the functional biology of organisms for the improvement of qualitative traits (4) and the opportunity to assemble comprehensive and genome-scale biochemical pathway networks (5, 6). The transition of traditional biology into an information-based science is marked by the need to efficiently store and manage data and to extract meaningful biological insights. This requires the development of annotation common standards, as well as methods for exchanging data across a broad range of organisms (7, 8).

Concomitant with the increasing ease of access to new genomes and the need for functional interpretation of Omics data, the number of metabolic databases has also expanded. Reactome (9) and Ingenuity Pathway analysis (www.ingenuity.com), e.g. integrate mostly animal and human pathways, while the Kyoto Encyclopedia of Genes and Genomes (KEGG) (10) aims to be a universal metabolic database in which pathways are organized into large networks using rigid map-like pathway schemes accommodating a multitude of biological processes from various organisms (11, 12). Probably one of the most comprehensive database system is the Pathway Tools suite. It provides tools for storing gene, enzyme and chemical compound data, and for conceptually organizing these into reactions, pathways, superpathways and organism level networks. It also includes tools for functional analysis and visualization, such as Omics data painting on the cellular overview (13, 14), metabolic flux analysis (15) and the querying capability for metabolic networks (16, 17). Species-specific metabolic databases, called Pathway/Genome Databases (PGDBs) for short, can be built automatically by matching annotated genes for a given species against a reference database of pathways. These derivative databases are ranked, according to the level of expert annotation they received, into three tiers. Tier 3 databases are generated purely computationally and are not curated in any way, tier 2 databases are partially curated and the highest level, tier 1, represents continually updated and intensively manually curated databases (18).

The main reference database is MetaCyc, which is built and maintained by the group of Peter Karp at SRI International, who also develops the Pathway tools software; the group has also developed extensive guidelines for the curation of primary and specialized metabolism (http://www.metacyc.org/) (19). MetaCyc contains metabolic reference pathways, which have been extracted from the literature by experts and as such represents an experimentally validated, universal repository for metabolic information across all realms of life (18). MetaCyc serves both as a repository of knowledge and as a reference database for the computational prediction of species-specific databases for organisms with annotated genomes (20). To date, about 9400 such derivative PGDBs have been generated and made available in the BioCyc database collection (http://biocyc.org/), but very few have been curated in a way that would corroborate the predicted metabolic network and integrate experimental information from the published scientific literature (16, 18). MetaCyc has become the de-facto standard in species-specific database curation, and Pathway Tools generated and manually curated PGDBs have been created across the domains of life, including bacteria [*Escherichia coli* in EcoCyc (21)], fungi (*Saccharomyces cerevisiae* in YeastCyc http://yeast.biocyc.org/), mammals [*Bos taurus* in CattleCyc (22)] and plants such as *Arabidopsis thaliana* in AraCyc (23, 24), *Medicago truncatula* in MedicCyc (25), *Fragaria vesca* in FragariaCyc (26), *Oryza sativa* in RiceCyc (27) and *Zea mays* in MaizeCyc (28). While most PGDBs are created at the species level, this is not required and they can be generated at arbitrary levels in the taxonomic hierarchy. For instance, PlantCyc is a kingdom-level database, which was built using plant-specific pathways from MetaCy (http://www.plantcyc.org/), as well as curated pathways of AraCyc, RiceCyc, and MedicCyc. PlantCyc has been continually expanded by adding new curator-approved plant pathways and used as a supplementary reference for predicting plant-specific databases, which constitute the Plant Metabolic Network (29).

Species-specific databases of the nightshade family can be found on the SolCyc site (http://solcyc.solgenomics.net/), which is hosted at the Sol Genomics Network (SGN), a comparative repository for a broad range of biological information revolving around species of the *Solanaceae* family (30–32).

The *Solanaceae* are a family of worldwide distribution characterized by its huge diversity. It consists of 3000–4000 species from about 100 genera prospering in very diverse habitats (33). The amazing biodiversity in the nightshade family is the result of a phylogenetic process that started millions of years ago (34). The stem age of the *Solanaceae* is estimated at approximately 49 million years ago. The split of two clades of the *Solanaceae*, i.e. *Solanum*, hosting almost half of the total species, and *Nicotiana* has been dated to circa 24 million years ago (35). Many of the world’s major crop species such as potato, tomato, eggplant, pepper and tobacco reside in the *Solanaceae* family, but members of the *Solanaceae* family are also widely used in the ornamental plant business (30) and as a valuable source for specialized metabolites of potential pharmaceutical importance (36, 37). Moreover, species of the *Solanaceae* have been a long-standing subject in classical and molecular genetic research (38), and some have become basic biological model systems, for instance, tomato in fruit development and maturation (39), petunia in the molecular genetics of flower development (40), tobacco in somatic cell genetics (41) and the black nightshade (*Solanum nigrum*) as ecological expression system (42).

Here, we describe several new additions to SolCyc. The first, SolanaCyc, is a PGDB specific to the family of the nightshades, which contains only experimentally determined pathways extracted from the scientific literature. The main purpose of this database is to integrate manually curated data into the metabolic networks of *Nicotiana* and *Solanum* specific databases and to serve as reference on the biochemistry and molecular biology in *Solanaceae* species. The second, NicotianaCyc, aims to be a comparative resource for metabolic pathways specific to the genus *Nicotiana*. Finally, four organism-specific databases, NtabacumCyc, NbenthamianaCyc, NsylvestrisCyc and NtomentosiformisCyc are aimed at providing tier 2-level metabolic information on key *Nicotiana* species. Consequently, the metabolic databases available in SolCyc have increased in both number and curation quality. SolCyc functions as a management hub for manual data curation and distribution within *Solanacea*-specific PGDBs. This setup ensures ongoing updates for those databases, which sets them apart from the overwhelming number of tier 3 PGDBs, constituting 99.5% of all existing PGDBs.

In the following, we describe the manual curation of new *Solanaceae*-specific pathways, the updating of existing MetaCyc pathways and the pathway validation in NtabacumCyc. We also discuss commonalities and differences of our database collection with other metabolic databases. The rationale for upgrading species-specific databases of the *Solanaceae* family is threefold: (i) to improve the completeness and accuracy for metabolic networks in important crop species such as tomato, potato and tobacco; (ii) to increase the reliability for analysis tools available in the databases for functional ‘Omics’ datasets; and (iii), as a long-term goal, to identify overlapping metabolic areas between *Solanaceae* host plants and pathogenic organisms as potential targets for concerted metabolic responses.

## Materials and methods 

### *Nicotiana* genomes reannotation

The assemblies for *N.tabacum* accession TN90 (GCA_000715135.1), *N.sylvestris* (GCF_000393655.1) and *N.tomentosiformis* (GCF_000390325.2) were downloaded from NCBI assembly (https://www.ncbi.nlm.nih.gov/assembly) (A. Bombarely, unpublished results) Previous mRNA annotations for each of the genomes were downloaded from SGN database (ftp://ftp.solgenomics.net/genomes/). Additionally mRNA sequences were complemented with publicly available Sanger ESTs from NCBI GenBank and assembled 454 ESTs from SGN (ftp://ftp.solgenomics.net/transcript_sequences/by_experiment/decipher_ntab/assembly/) (78). De-novo repeats were analyzed using RepeatModeler v1.0.8 (default parameters). De-novo repeats and mRNA were used to re-annotate the *Nicotiana* genomes using Maker-P (79) with the default parameters. A total of 72 866, 37 162 and 36 509 gene models and 69 211, 35 553 and 34 378 protein coding genes were annotated for the *N.tabacum, N.sylvestris* and *N.tomentosiformis* genomes, respectively. Functional annotation was performed searching annotated proteins by sequence similarity using BlastP (with a hit e-value cutoff < 1e-20) of the coding protein genes with the GenBank NR, TAIR10 and SwissProt databases (downloaded on the 21 July 2014). Additionally the protein domains were annotated using InterProScan. Functional annotations were integrated using the program AHRD v2.0.2 (https://github.com/groupschoof/AHRD).

### Database setup and curation

The SolCyc collection of databases for *Solanum* and *Nicotiana* specific databases are assembled on the Pathway Tools curator GUI which connects the MetaCyc database via VPN to the internal MYSQL server at SRI International and the curatable *Solanaceae* MYSQL and FILE databases via SSH to the internal development site at SGN. Curation is done by extracting information about pathways, reactions, genes, enzymes and compounds from peer-reviewed resources, for instance, NCBI’s PubMed (https://www.ncbi.nlm.nih.gov/pubmed), Google scholar (https://scholar.google.com/), Nomenclature Committee of the International Union of Biochemistry and Molecular Biology (NC-IUBMB) (http://www.chem.qmul.ac.uk/iubmb/enzyme/), UniProt and Swiss-Prot (http://www.uniprot.org/), GenBank (https://www.ncbi.nlm.nih.gov/genbank/), the Gene Ontology Consortium (http://www.geneontology.org/) and chemical databases such as ChEBI (http://www.ebi.ac.uk/chebi/), PubChem (https://www.ncbi.nlm.nih.gov/pccompound) and ChemSpider (http://www.chemspider.com/). The Pathway Tools predicted metabolic networks for species-specific PGDBs are evaluated, falsely predicted pathways removed and new pathways for the respective species added. Newly curated pathways are assembled in accordance with the published literature and reactions furnished with full or partial EC numbers, catalyzing enzymes, encoding genes and regulatory interactions. Hyperlinks to pathways feeding into or branching out of the pathway are provided and comments written to pathways, genes and enzymes. BLAST of UniProt enzymes allows for matching and merging with gene and protein ID’s of the individual species-specific database.

## Results and discussion

### The SolCyc management of *Solanaceae* databases

**Figure 1.**
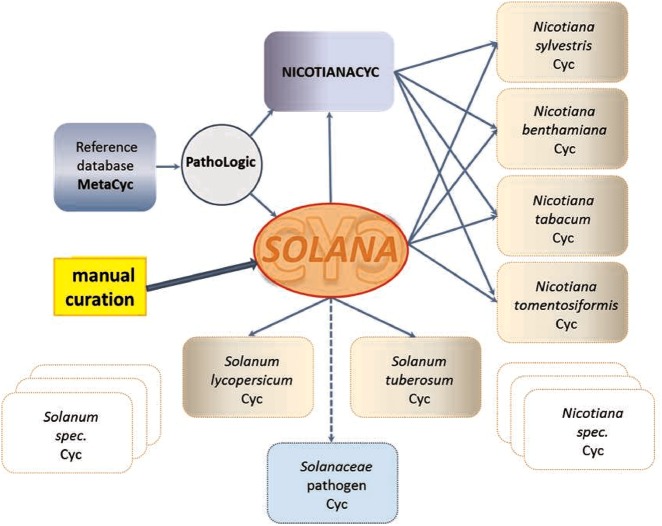
Creation and curation flux of SolanaCyc (explanation see text).

SolCyc currently provides access to five PGDBs of *Solanaceae* species (tomato, potato, pepper, petunia and tobacco) and one PGDB of the *Rubiaceae* family, i.e. *Coffee* species. All SolCyc databases at the SGN have been created using the Pathway Tools software component Pathologic, which predicts the metabolic networks in those organisms based on MetaCyc as reference database (43). Other tools were used for these databases to transform GFF (Generic Feature Format) annotation files to the files that Pathway Tools parse (https://github.com/solgenomics/cyctools). The freely accessible SolCyc databases represent computational predictions of their metabolic maps and have not been reviewed or updated by curators. The recent introduction of the curated SolanaCyc database aims to elevate the two Solanum databases and four *Nicotiana* databases (Figure 1) to the tier 2 class of curated databases, and in the near future to the tier 1 category, i.e. the tier that represents the most highly curated databases (18).

**Figure 2.**
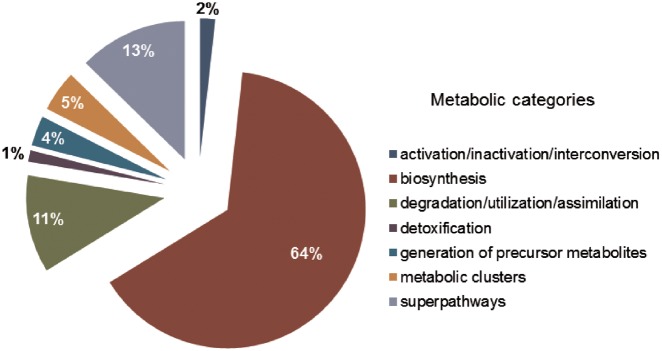
SolanaCyc pathway breakdown into metabolic categories.

SolanaCyc was created by pathologic as a MYSQL database. Curated pathways from MetaCyc associated with the taxonomic range of the *Solanaceae* family were imported into SolanaCyc and complemented with newly curated biochemical pathways. Except for new compounds that were created in MetaCyc to stay compatible with MetaCyc’s compound identifier system and curation standard, new pathways, genes and enzymes from *Solanaceae* species were curated into SolanaCyc. The manually updated and verified SolanaCyc pathways were subsequently distributed to the genus-specific NicotianaCyc database and the individual *Solanum* and *Nicotiana* specific databases. NicotianaCyc was established as a database that collects curated metabolic data pertaining only to *Nicotiana* species, hence developing a database with a very high specificity and relevance towards this plant genus. SolanaCyc’s curation flow also applies to future PGDBs, for instance, databases for the metabolism of pathogens known to affect *Solanaceae*, or any new *Solanaceae* species for which an annotated genome has been published (Figure 1). The current version of SolanaCyc (1.0) contains 199 pathways, which are comprised of 835 reactions and 1441 compounds (Table 1), including 30 new pathways which have been extracted, curated and added to the database in 2016 and the re-evaluation and updating of imported pathways from MetaCyc with pertinent metabolic data. The distribution of pathways among metabolic categories in SolanaCyc (Figure 2) is similar to the breakdown of pathways observed in MetaCyc and PlantCyc (Supplementary Figure S1 A and B). That is not surprising given that PlantCyc was initiated using all of MetaCyc’s plant pathways as resource and much of SolanaCyc’s metabolic content has been imported from MetaCyc. In all three databases, biosynthetic pathways are the most prevalent, followed by pathways involved in the degradation or utilization of compounds. However, the number of degradation pathways in MetaCyc is 12 and 17% higher in comparison to the corresponding pathway numbers in PlantCyc and SolanaCyc. This is very likely a reflection of the curation priority in MetaCyc, which is more focused on microbial metabolism where degradation pathways dominate biosynthesis pathways in a 60:40 ratio. In addition to the 199 pathways, SolanaCyc lists 29 superpathways defined as pathways that contain at least one base pathway, i.e. one of the 199 pathways in SolanaCyc, and additional pathways and/or reactions. Superpathways are useful to highlight larger sections of metabolism and emphasize links to interconnecting-related pathways (44).

**Table 1.** Summary of the numbers of pathways, enzymatic reactions, enzymes, genes and compounds contained in SolanaCyc (version 1.0)

	SolanaCyc
Pathways	199
Superpathways	29
Enzymatic reactions	835
Enzymes	257
Protein complexes	35
Genes	209
Compounds	1441

Currently, SolanaCyc hosts 27 species from 9 genera of the *Solanaceae* family for which pathways with experimentally verified data have been curated (Table 3). The species with the highest number of manual curated pathways are tomato (87 pathways), tobacco (72 pathways), potato (56 pathways), petunia (25 pathways), pepper (14 pathways) and the wild tomato species *Solanum habrochaites* (12 pathways). Metabolic databases with a high degree of manual curation and continuous updating are rare and mostly limited to model organisms, for instance, *Arabidopsis thaliana* in AraCyc (23) or species with high impacts on human nutrition (26) and health (Supplementary Table S1). The latter involves two databases that focus on parasitic protozoans known to cause sleeping sickness (*Trypanosoma brucei*) and the skin affecting Leishmaniasis disease (*Leishmania major*). Both TrypanoCyc (45) and LeishCyc (46) are MetaCyc derived databases, which are characterized by a significant degree of manual curation and counted among the tier 1 category of BioCyc databases.

### Pathway curation in SolanaCyc

**Figure 3.**
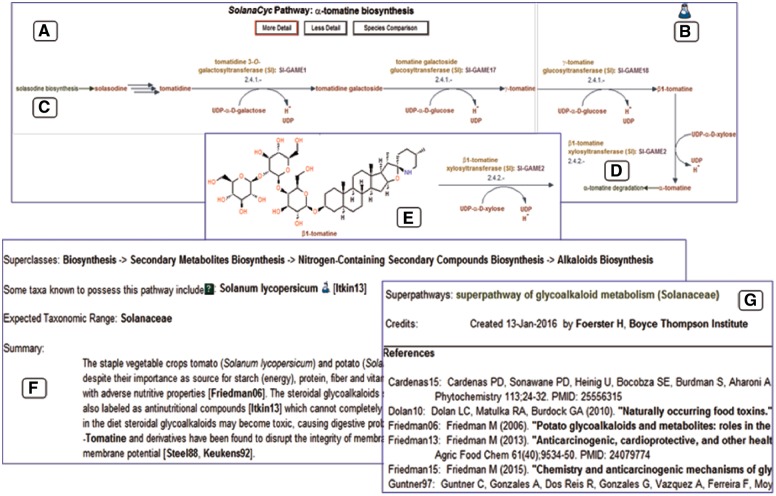
Representation of the ?-tomatine biosynthesis in SolanaCyc. (**A**) Pathway diagram. (**B**) Indicative evidence for the curation status of the pathway. Hyperlinks to related pathways that either feed into (**C**) or branch out (**D**) of the pathway. (**E**) Structure of pathway compounds after increasing the detail of the pathway. (F) Pathway summary with (**G**) corresponding literature that is linked to external databases (see also text).

Manual curation of metabolic databases is a time and labor intensive endeavor, which requires curators with a biochemical and molecular biology background (biocurators) and a high familiarity with the database’s structure, features and performance. The extraction and editing of data from peer-reviewed publications are a central element in the multifaceted curation procedure. Reported details about catalyzing enzymes such as physico-chemical properties and kinetic parameters, cellular localization and regulation as well as encoding genes and their expression pattern is noted on the respective detail pages and summarized in enzyme and gene specific comments. These comments also point out the employed purification method and status of the characterized enzyme and reference all relevant publications. FASTA files of enzymes or genes deposited in GenBank and/or UniProt are blasted against the PGDB of the species and result in the merge of the curated enzymes and genes with the annotated genes and enzymes of the species database. The next curation steps concern the designation of the reaction and built of the pathway. If reported, reactions are associated with full or partial EC numbers, which are either obtained from the curated literature or inferred by the curator using external resources (listed under Material and Methods). The general process of building a pathway with a more detailed description of the pathway curation is depicted in Figure 3. The outlined curation process applies to both new and revised pathways, with the latter focusing on adding species-specific genes and enzymes or reactions complementing existing pathway sequences. Manual curation of all elements defining a pathway, i.e. enzymes, genes, reactions and compounds significantly increases the accuracy of metabolic routes and in return diminishes the display of false positives in the database.

During the curation process, biocurators face a number of challenges which have to be solved in accordance with the rules and structural setup of the database. Several decisions a biocurator has to make when adding a new pathway require intensive study of the relevant literature. MetaCyc and its derivative databases such as SolanaCyc define pathways and their boundaries in a different way than KEGG. The modular concept of Cyc pathways describes base pathways for single organisms with a defined start and stable end metabolite, the latter usually a branch-point toward other related base pathways. This kind of pathway, which would ideally have been experimentally verified for individual species, is referred to as conspecific pathway. Pathways composed of information from various species are chimeric pathways, which imply the notion that this pathway may not occur in all of those species (11, 44). However, it is not at all common that all catalyzing enzymes and genes of a pathway have been studied in any one single organism, especially not in higher organisms such as plants. Consequently, a number of conspecific pathways may in all probability occur in all the species contributing metabolic information. Likewise, a tricky decision is the definition of the taxonomic range of a pathway. In particular, specialized metabolites typically found only in confined plant lineages might be reported in later publications in other species due to improved analysis tools or the incentive to look specifically for those compounds. However, the dynamic setup of the Pathway tool software ensures the integration of relevant data in new database releases should new pertinent literature have become available.

Meaningful mining and analyses of the many data in PGDBs of the MetaCyc family very much depend on the quality of the data and the available bioinformatics tools and applications in the database (47). Although efforts have been undertaken to support the work of biocurators with automated text mining tools, for instance (48), the extraction of essential information from the published literature, its translation into validated data and the supervision of the subsequent transformation and visualization of this knowledge in the functional blueprint of an organism is still generally performed by biocurators. The number of errors in databases curated by biocurators is much lower than in semi-automated approaches to extract information from the literature. This is based on the unique ability of professional curators to comprehend and analyze intricate contexts and critically assess inconsistent conclusions (49).

The *Solanaceae* family is known to produce a number of specialized metabolites, also referred to as secondary metabolites, such as alkaloids, flavonoids and polyphenols which have been employed in varying capacities for human health and food. Tomatoes with elevated levels of healthy polyphenolic phytochemicals exerting antioxidative activities (50, 51) and cancer-preventive properties (52), as well as tobacco used for the production of plant-derived pharmaceuticals (53) and recombinant interferons (54) have already been exploited for that reason. Plant-specific metabolites are typically produced in lineage-specific manners, which are often a result of convergent evolution in the various species (55). Some of those specialized metabolites, namely acylsugars and certain steroidal glycoalkaloids such as α-chaconine and α-solanine (*S.tuberosum*) or solasodine and α-tomatine (*S.lycopersicum*) are biosynthesized by and restricted to members of the nightshade family. We have curated those pathways from the literature, added them to SolanaCyc and use the biosynthesis of the glycoalkaloid α-tomatine in tomato as an example to demonstrate data processing and display.

Except for the first step in the pathway, the conversion of solasodine to tomatidine, all enzymes and encoding genes catalyzing the reactions in the α-tomatine biosynthesis in *S.lycopersicum* have been experimentally characterized and described in the literature (56, 57). The resulting pathway diagram (Figure 3A) shows the conversion sequence of compounds, colored in red. The reactions are furnished with EC numbers, reaction partners, enzymes (in yellow) and genes (in magenta). An evidence icon provides information about the curation quality of the pathway, i.e. computationally predicted (computer icon) versus experimental evidence (flask icon) (Figure 3B). Pathway names colored in green provide hyperlinks to pathways that either feed into (Figure 3C) or branch out to related pathways (Figure 3D). The level of detail of the pathway can be changed; increasing the detail reveals the structure of the involved compounds (Figure 3E). The most important facts about the pathway are provided in the pathway summary, outlining the general significance of the pathway but also discussing more specific, pathway related issues such as rate-limiting steps, key-enzymes and regulation or stereochemistry of involved compounds (Figure 3F). This information is extracted from the scientific literature cited in the reference list (Figure 3G). Each of the pathway elements such as enzymes, genes, reactions and compounds has its own detail page where in-depth information about the subject, unification links and relationship links to external databases are available.

### Pathway validation: NtabacumCyc

The SolCyc family of PGDBs has been recently extended by four *Nicotiana-*specific databases, i.e. NtabacumCyc (*Nicotiana tabacum*), NbenthamianaCyc (*Nicotiana benthamiana*), NsylvestrisCyc (*Nicotiana sylvestris*) and NtomentosiformisCyc (*Nicotiana tomentosiformis*). The databases were constructed by using genomic annotation information of the respective *Nicotiana* species (58, 59) as input for the pathologic component of pathway tools, which assembled the databases using MetaCyc as reference database. The database statistics for the four *Nicotiana* species (Table 2) reflects their genetic makeup. The two allotetraploid species *N.tabacum* and *N.benthamiana* display a considerably larger number of predicted genes and associated enzymes, whereas the two diploid species *N.sylvestris* and *N.tomentosiformis*, the ancestral parents of *N.tabacum* (60), have an accordingly smaller number in the predicted gene and enzyme category.

**Table 2.** Summary of the predicted numbers of pathways, enzymatic reactions, enzymes, genes and compounds in the Cyc’s of *Nicotiana tabacum*, *Nicotiana tomentosiformis*, *Nicotiana sylvestris*, and *Nicotiana benthamiana*^a^

	Nicotiana	Nicotiana	Nicotiana	Nicotiana
	*tabacum*	*tomentosiformis*	*sylvestris*	*benthamiana*
Pathways	569	517	449	541
Enzymatic reactions	3309	2907	2659	3008
Enzymes	19 517	9257	6346	12 506
Genes	69 211	34 378	35 533	57 139
Compounds	2424	2100	1981	2163

a The pathway numbers refer only to base pathways of the species and do not include superpathways.

**Figure 4.**
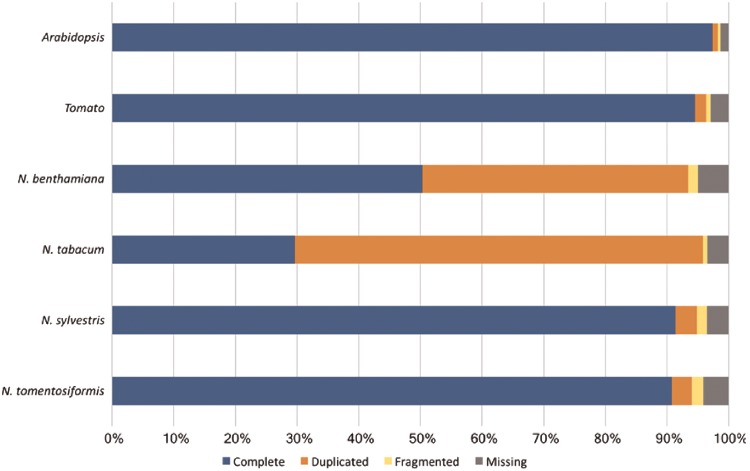
Assessment of the Nicotiana genome completeness. The percentages of the 1440 embryophyta single-copy orthologs identified in one complete copy (Complete), more than one complete copy (Duplicated), partially (Fragmented) or not identified (Missing) are shown.

**Figure 5.**
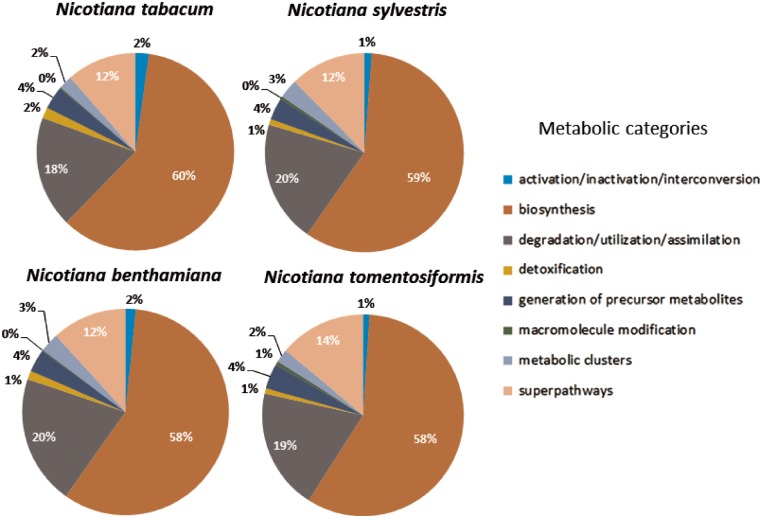
Distribution of predicted pathways across the various compound classes in the Cyc's for *Nicotiana tabacum, Nicotiana sylvestris, Nicotiana benthamiana, and Nicotiana tomentosiformis*.

However, it is noticeable that for *N.sylvestris* clearly fewer pathways, enzymatic reactions and enzymes have been predicted than for *N.tomentosiformis*, despite the fact that the difference in gene count and compound numbers are not significant (Table 2). The reason for that may lie in two linked input parameters, which have significant bearing on the outcome of the Pathologic prediction. The first parameter is the metabolic completeness of the reference database. Pathologic can only predict pathways, which already exist in the reference database, i.e. MetaCyc. If the *Nicotiana* species, including *N.sylvestris*, have pathways that have not been curated in MetaCyc, the metabolic networks are less accurately reflected and potentially differ from each other to a larger extent. Pathologic takes the annotated set of enzymes to infer all possible reactions (reactome), which in turn is used to predict the pathways in the organism’s metabolic network (61). The number of predicted pathways directly depends on the number of predicted enzyme functions. Consequently, the lower numbers of predicted enzymes and enzymatic reactions in *N.sylvestris* in comparison to *N.tomentosiformis* results in the reduction in predicted pathways in NsylvestrisCyc. This relationship is confirmed by the ratio between enzymatic reactions and pathway predictions within the *Nicotiana* species, which is fairly constant and lies between 5.6 reactions per pathway (*N.benthamiana*) and 5.9 reactions per pathway (*N.sylvestris*). The second input factor of importance for Pathologic is the quality of the genome assembly and annotation, which primarily defines the number of genes, enzymes and enzymatic reactions. Assessing the genome completeness of these four *Nicotiana* species using BUSCO (62) with the Embryophyta dataset identified complete copies of about 95% of the 1440 single-copy orthologs in each of the genomes. These levels of completeness are similar to that of tomato and slightly lower than that of *Arabidopsis* (Figure 4). Despite the differences in ploidy and the number of predicted enzymes and genes between the *Nicotiana* species the resulting composition of metabolic networks deviates only marginally from each other. The distribution of pathways in the various metabolic classes is very similar, which may indicate that the basic setup of metabolism has not dramatically changed between the studied *Nicotiana* species (Figure 5).

Despite the good agreement for the composition of the metabolic networks each *Nicotiana* species also deviates from each other by employing sets of pathways which have only been predicted for the individual organism (Supplementary Figure S2) (63). The overlap section of predicted pathways common to *N.tabacum* and its parental species *N.tomentosiformis* and *N.sylvestris* adds up to 73.2% of all pathways. Among the three species *N.tabacum* has the highest percentage of pathways (9.9%) outside the common intersection, followed by *N.tomentosiformis* (0.6%) and *N.sylvestris* (0.4%). The comparison of predicted pathways between each two individual *Nicotiana* species (results not shown) revealed the highest divergence between *N.tabacum* (23%) and *N.sylvestris* (1.3%). This is expected considering that pathologic predicted 120 fewer pathways for *N.sylvestris* than for *N.tabacum*, whereas *N.tomentosiformis* was only 28 pathways short of the pathway count predicted in *N.tabacum*. Out of the 67 unique *N.tabacum* pathways, the main part, i.e. 27 (∼40%) pathways, was classified under the specialized metabolite category. The divergence in some metabolic divisions in cultivated and wild tobacco species is certainly also due to the observed high molecular diversity in the genus *Nicotiana*, where the high degree of genetic polymorphism results in gaining or losing the ability to biosynthesize a range of metabolites (64).

After in-depth manual validation of the 569 predicted pathways in NtabacumCyc, 156 pathways were considered invalid (Supplementary Table S2). The validation process included the search for confirmation of corresponding genes, enzymes and reactions in *N.tabacum* in various external databases such as the EC nomenclature, GenBank and UniProt. In addition, NCBI’s PubMed and Google scholar were used to find relevant literature either validating or rejecting the occurrence of pathways or components thereof in *N.tabacum*. Of the 156 invalid pathways 112 pathways were found to be specific for bacteria, fungi or metazoa with no evidence to occur in tobacco. Furthermore, 41 pathways of specialized metabolites were identified which have not been reported in this species. The constant curation and updating of pathways in MetaCyc often results in deleting obsolete or redundant pathways from the database (18). Four pathways which are no longer present in MetaCyc but had been predicted with the previous release version were also labeled as invalid pathways in NtabacumCyc. On the other hand, pathways, which did not fit the taxonomic range of *N.tabacum* but for which evidence pointing to their presence could be found, were kept as pathway variants, defined as pathway routes that deviate from the described reaction sequence but achieve the same metabolic goal (43). Variant pathways will stay in the database until research confirms or rejects the presence of these variants in the PGDB.

### The future direction of SolCyc

Because the *Solanaceae* have a tremendous impact on the daily life of humans they have been systematically explored for a long time. The SGN’s role as a center for biological information around the *Solanaceae* necessitates to efficiently manage the ever-increasing amounts of data. That also includes the biochemical pathways of selected *Solanaceae* species which are stored as a collection of PGDBs in the SolCyc database at SGN.

With the establishment of manually curated taxon-specific databases, the family-specific SolanaCyc and the genus-specific NicotianaCyc, a new phase for SolCyc has started which allows curating and enriching *Solanum* and *Nicotiana* specific databases with a increased level of detail. Unlike MetaCyc, in SolCyc only metabolic data relevant to members of the nightshade family are curated. In comparison to PlantCyc, which concentrates on creating new plant-specific PGDBs and curating selected aspects of plant metabolism, SolanaCyc represents an actively curated database, whose main goal it is to add new *Solanaceae* pathways and missing *Solanaceae* specific information to existing pathways in the database. The main purpose of SolanaCyc is growing into a database which accommodates the most complete collection of metabolic data on the *Solanaceae*, hence making SolanaCyc a knowledgebase for the biochemistry of this plant family. The features of MetaCyc derived databases facilitate comparative analysis between species for which Cyc’s have been created, which will make SolCyc a useful tool for comparative systems biology within and beyond the *Solanaceae* family.

The value of the metabolic databases generated at SGN has already been demonstrated by exploiting the resources of the database for the elucidation of the capsaicinoid biosynthesis in pepper. Predicted pepper transcripts obtained from capsaicinoid producing tissues were integrated and annotated in the SGN database and translated into a pepper-specific PGDB (CapCyc) allowing for the visualization of the pathway and related metabolic processes on the metabolic map of *Capsicum annuum* (65). The development of evidence-based metabolic networks has been proven beneficial for studying species metabolism in genome-scale studies addressing crop yield in maize (66), stress-related changes and regulation in the biosynthesis of essential amino acids and phytohormones in rice (67) and even microbial community metabolism in environmental PGDBs (68).

The deep exploration of primary and specialized metabolism in plant genera such as *Solanum* and *Nicotiana* has elucidated the complex interrelationships between the plant and its environment. The *Solanaceae* produce a great variety of specialized compounds, amongst them a number of alkaloids used by the defense system of the plant to repel pathogens and harmful insect attacks (69, 70). The long-term goal at SGN is to develop a PathogenCyc with the intention to collect metabolic data on pathogenic organisms infesting *Solanaceae*. Similar attempts on single pests have already been undertaken to determine the common metabolic denominator and to identify metabolic pathways used as gateway by the attacker to gain access to the host. The analytical power of MetaCyc derived databases has been used in RiceCyc for the identification of metabolic differences in rice plants susceptible or resistant against a common pest in rice, the small brown planthopper (71). Another example for host–pathogen interactions is the citrus greening disease threatening a large proportion of the citrus production worldwide. This disease is even more complex as it is caused by a bacterial pathogen which lives in symbiosis with an insect, the Asian citrus psyllid. The psyllid serves as vector and transmits the pathogen to the host plant. The study of the proteomes of insects either carrying or not carrying the bacterial pathogen revealed differences in the protein spectrum which is a first step towards deciphering the metabolic interplay between host plant, bacterial pathogen and insect symbiont (72).

The approach of extending the central role of SolCyc as organizational hub for SGN metabolic databases and SolanaCyc as the curator database for the *Solanaceae* family was chosen to use synergies in the current database infrastructure, which will guarantee the most intensive and complete curation of *Nicotiana* and *Solanum* specific PGDBs, the upgrade of the databases to tier 1 and the creation of a database environment that allows species comparison and identification of common intersections of metabolism between *Solanaceae* species and pathogenic organisms.

## Conclusions

It is well documented that the accuracy of computationally predicted PGDBs is directly correlated with the quality of the genome annotation of a species (61). The *Solanaceae* family comprises a number of important crop plants, which brought this plant family into the focus of breeders, geneticists, biochemists and bioinformaticians. High quality annotated and constantly updated genomes like that of *Solanum lycopersicum* have provided insights in many aspects of metabolism including neofunctionalization of genes for qualitative traits such as color and flavor (73). The use of new sequencing technologies has propelled forward functional genomics in tomato by improving the reference sequence (4), which in turn advances the exploitation of large-scale proteomics and metabolomics data sets for the identification of new proteins and encoding genes (74). Other genomic databases across the spectrum of organisms have also shown that the improvement of annotation is fundamental for understanding principal functions encrypted in the genome. The analysis and annotation of the genome from the fungus *Aspergillus westerdijkiae* allowed for the identification of genes encoding for enzymes involved in host invasion and pathogenicity (75). Manually curated genome databases for cyanobacteria and rhizobia, i.e. CyanoBase and RhizoBase, have enabled a better access and display to functions and products of genes in the databases (76) and the OrchidBase database holds the information of expressed sequences of orchid flowers permitting the search for unigenes in these transcriptomes (77).

With its 72 manually curated pathways (Table 3), *N.tabacum* is next to *S.lycopersicum*, the most highly curated plant species in SolanaCyc contributing about one third of all pathways in the database (Supplementary Figure S3). *N.tabacum* is a valuable crop and model organism and has been the subject of numerous biochemical, genetic, molecular biological and bioinformatics research projects. However, although its genome has been closely studied and improved (58, 59) a finished genome sequence has yet to be obtained. It is to be expected that with the continuous efforts to enhance the annotation quality of the *Nicotiana* species genomes and the enrichment of curated metabolic data in the associated databases will contribute to a more accurate representation of their metabolic networks.

**Table 3.** List of *Solanaceae* species, which contributed validated experimental data to pathways in SolanaCyc^a^

Genus/Species	Number of pathways
Solanum	
*Solanum lycopersicum*	87
*Solanum tuberosum*	56
*Solanum habrochaites*	12
*Solanum pennellii*	8
*Solanum aculeatissimum*	2
*Solanum melongena*	2
Nicotiana	
*Nicotiana tabacum*	72
*Nicotiana attenuata*	7
*Nicotiana sylvestris*	5
*Nicotiana benthamiana*	2
*Nicotiana langsdorffii x Nicotiana sanderae*	1
*Nicotiana plumbaginifolia*	1
*Nicotiana rustica*	1
*Nicotiana suaveolens*	1
Cestrum	
*Cestrum elegans*	1
Petunia	
*Petunia x hybrida*	25
Capsicum	
*Capsicum annuum*	14
*Capsicum baccatum*	1
*Capsicum chinense*	2
*Capsicum frutescens*	1
Anisodus	
*Anisodus acutangulus*	2
Atropa	
*Atropa belladonna*	5
Hyoscyamus	
*Hyoscyamus albus*	3
*Hyoscyamus muticus*	1
*Hyoscyamus niger*	4
Datura	
*Datura inoxia*	1
*Datura stramonium*	6

a Note that pathways are associated with more than one species.

## Supplementary data

Supplementary data are available at *Database* Online .

## Funding

This work was supported by PMI.

*Conflict of interest*. None declared.

## Supplementary Material

Supplementary Data
