# Supplementary material for: SolCyc: a database hub at the Sol Genomics Network (SGN) for the manual curation of metabolic networks in Solanum and Nicotiana specific databases
Source: Database (Oxford). 2018 May 10;2018:bay035. doi: 10.1093/database/bay035 (PMC5946812; doi:10.1093/database/bay035)
Supplement: Supplementary Data [file bay035_supp.zip › tab 1 S.docx]

| Database | Version | Taxonomic range | # of base pathways | # of superpathways | % of pathways with experimental evidence | source |
| --- | --- | --- | --- | --- | --- | --- |
| MetaCyc | 20.1 | All domains of life | 2492 | 380 | 96.8 | MetaCyc |
| SolanaCyc | 1.0 | Solanaceae | 199 | 29 | 89.0 | SGN |
| PlantCyc | 11.0 | *Viridiplantae* | 1094 | 119 | 79.4 | PMN |
| NicotianaCyc | 1.0 | *Nicotiana* | 72 | 13 | 74.1 | SGN |
| AraCyc | 14.0 | *Arabidopsis thaliana* | 542 | 77 | 62.8 | PMN |
| SoyCyc | 7.0 | *Glycine max* | 531 | 65 | 18.1 | PMN |
| CornCyc | 7.0 | *Zea mays* | 484 | 61 | 15.4 | PMN |
| NtabacumCyc | 1.0 | *Nicotiana tabacum* | 446 | 58 | 14.3 | SGN |
| ChlamyCyc | 6.0 | *Chlamydomonas reinhardtii* | 329 | 44 | 12.6 | PMN |
| PoplarCyc | 9.0 | *Populus trichocarpa* | 535 | 64 | 8.5 | PMN |
| MaizeCyc | 2.2.19 | *Zea mays* | 424 | 55 | 8.1 | Gramene |
| RiceCyc | 3.3.1.2.19 | *Oryza sativa* | 308 | 50 | 6.7 | Gramene |
| GrapeCyc | 6.0 | *Vitis vinifera* | 495 | 62 | 5.7 | PMN |
| OryzaCyc | 4.0 | *Oryza sativa* | 483 | 60 | 5.2 | PMN |
| PapayaCyc | 5.0 | *Carica papaya* | 475 | 63 | 3.3 | PMN |
| CassavaCyc | 6.0 | *Manihot esculenta* | 503 | 64 | 2.1 | PMN |
| TomatoCyc | 2.0 | *Solanum lycopersicum* | 518 | 70 | 2.0 | PMN |
| MossCyc | 5.0 | *Physcomitrella patens* | 473 | 62 | 1.3 | PMN |
| PotatoCyc | 3.0 | *Solanum tuberosum* | 493 | 65 | 1.3 | PMN |
| SorghumBicolorCyc | 4.0 | *Sorghum bicolor* | 477 | 63 | 1.1 | PMN |
| BarleyCyc | 4.0 | *Hordeum vulgare* | 478 | 62 | 0.9 | PMN |
| ChineseCabbageCyc | 4.0 | *Brassica napa ssp. pekinensis* | 471 | 62 | 0.4 | PMN |
| BrachypodiumCyc | 4.0 | *Brachypodium distachyon* | 470 | 61 | 0.2 | PMN |
| SetariaCyc | 4.0 | *Setaria italic* | 482 | 67 | 0.2 | PMN |
| SwitchgrassCyc | 4.0 | *Panicum virgatum* | 521 | 66 | 0.2 | PMN |
| MedicCyc | 16.0 | *Medicago truncatula* | 374 | 33 | ? | Noble |
| WheatACyc | 2.0 | *Triticum urartu* | 487 | 64 | 0 | PMN |
| WheatDCyc | 2.0 | *Aegilops tauschii* | 479 | 61 | 0 | PMN |
| SpirodelaCyc | 2.0 | *Spirodela polyrhiza* | 437 | 59 | 0 | PMN |
| SelaginellaCyc | 5.0 | *Selaginella moellendorffii* | 416 | 65 | 0 | PMN |
| LycoCyc | 3.3 | *Solanum lycopersicum* | 456 | 63 | 0 | SGN |
| PotatoCyc | 2.1 | *Solanum tuberosum* | 419 | 58 | 0 | SGN |
| PetCyc | 2.4 | *Petunia x hybrida* | 130 | 54 | 0 | SGN |
| BenthamianaCyc | 2.0 | *Nicotiana benthamiana* | 344 | 41 | 0 | SGN |
| CoffeaCyc | 2.4 | *Coffea sp.* | 312 | 39 | 0 | SGN |
