# Supplementary material for: SolCyc: a database hub at the Sol Genomics Network (SGN) for the manual curation of metabolic networks in Solanum and Nicotiana specific databases
Source: Database (Oxford). 2018 May 10;2018:bay035. doi: 10.1093/database/bay035 (PMC5946812; doi:10.1093/database/bay035)
Supplement: Supplementary Data [file bay035_supp.zip › table 2 S.docx]

| glycine betaine biosynthesis I (Gram-negative bacteria) |
| --- |
| glycine betaine biosynthesis V (from glycine) |
| histamine biosynthesis |
| diphthamide biosynthesis (archaea) |
| L-citrulline biosynthesis |
| protein citrullination |
| S-adenosyl-L-methionine cycle I |
| glycine biosynthesis II |
| glycine biosynthesis IV |
| L-asparagine biosynthesis III (tRNA-dependent) |
| L-citrulline-nitric oxide cycle |
| L-glutamate biosynthesis III |
| L-phenylalanine biosynthesis I |
| L-proline biosynthesis I |
| L-selenocysteine biosynthesis II (archaea and eukaryotes) |
| L-tyrosine biosynthesis IV |
| 2,3-dihydroxybenzoate biosynthesis |
| acetaldehyde biosynthesis I |
| CMP-3-deoxy-D-manno-octulosonate biosynthesis II (from D-arabinose 5-phosphate) |
| GDP-L-fucose biosynthesis II (from L-fucose) |
| glycogen degradation I |
| superpathway of UDP-glucose-derived O-antigen building blocks biosynthesis |
| UDP-α-D-glucuronate biosynthesis (from myo-inositol) |
| UDP-L-arabinose biosynthesis II (from L-arabinose) |
| peptidoglycan biosynthesis I (meso-diaminopimelate containing) |
| peptidoglycan biosynthesis III (mycobacteria) |
| coenzyme A biosynthesis II (mammalian) |
| lipoate salvage I |
| chlorophyllide a biosynthesis III (aerobic, light independent) |
| 1,4-dihydroxy-2-naphthoate biosynthesis II (plants) |
| menaquinol-6 biosynthesis |
| menaquinol-8 biosynthesis |
| menaquinol-9 biosynthesis |
| γ-glutamyl cycle |
| L-ascorbate biosynthesis V |
| arachidonate biosynthesis IV (8-detaturase) |
| cyclopropane fatty acid (CFA) biosynthesis |
| docosahexaenoate biosynthesis III (mammals) |
| fatty acids biosynthesis (yeast) |
| icosapentaenoate biosynthesis V (∆8 desaturase) |
| linoleate biosynthesis II (animals) |
| palmitoleate biosynthesis I (*E. coli*) |
| palmitoleate biosynthesis III (cyanobacteria) |
| stearate biosynthesis III (fungi) |
| 3-phosphoinositide biosynthesis |
| phosphatidate metabolism, as a signaling molecule |
| phosphatidylcholine biosynthesis V |
| phosphatidylserine biosynthesis II |
| cholesterol biosynthesis I |
| cholesterol biosynthesis II (via 24,25-dihydrolanosterol) |
| cholesterol biosynthesis III (via desmosterol) |
| lanosterol biosynthesis |
| superpathway of cholesterol biosynthesis |
| monoacylglycero metabolism (yeast) |
| ethylene biosynthesis III (microbes) |
| serotonin and melatonin biosynthesis |
| pyrimidine deoxyribonucleotides *de novo* biosynthesis III |
| mRNA capping I |
| tRNA methylation (yeast) |
| tRNA splicing |
| adenine and adenosine salvage IV |
| pyrimidine deoxyribonucleotides biosynthesis from CTP |
| eumelanin biosynthesis |
| L-dopachrome biosynthesis |
| autoinducer Al-1 biosynthesis |
| dehydroscoulerine biosynthesis |
| dhurrin biosynthesis |
| papaverine biosynthesis |
| sanguinarine and macarpine biosynthesis |
| apigeninidin 5-O-glucoside biosynthesis |
| epoxypseudoisoeugenol-2-methylbutyrate biosynthesis |
| flavonoid biosynthesis (in equisetum) |
| gentiodelphin biosynthesis |
| justicidin B biosynthesis |
| luteolinidin 5-O-glucoside biosynthesis |
| medicarpin biosynthesis |
| phenylpropanoids methylation (ice plant) |
| pterostilbene biosynthesis |
| quercetin sulfate biosynthesis |
| rose anthocyanin biosynthesis II (via cyanidin 3-O-β-D-glucoside) |
| rosmarinic acid biosynthesis II |
| salvianin biosynthesis |
| superpathway of anthocyanin biosynthesis (from delphinidin 3-O-glucoside) |
| superpathway of anthocyanin biosynthesis (from pelargonidin 3-O-glucoside) |
| ternatin C5 biosynthesis |
| kauralexin biosynthesis |
| oryzalide A biosynthesis |
| 1D-myo-inositol hexakisphosphate biosynthesis II (mammalian) |
| D-myo-inositol (1,3,4)-trisphosphate biosynthesis |
| inositol pyrophosphates biosynthesis |
| costunolide biosynthesis |
| linalool biosynthesis II |
| soybean saponin I biosynthesis |
| superpathway avenacin A biosynthesis |
| valencene and 7-epi-α-selinene biosynthesis |
| hypoglycin biosynthesis |
| salidroside biosynthesis |
| 2'-deoxymugineic acid phytosiderophore biosynthesis |
| enterobactin biosynthesis |
| glycerol degradation III |
| methylglyoxal degradation II |
| 4-aminobutyrate degradation II |
| 4-aminobutyrate degradation III |
| allantoin degradation to ureidoglycolate I (urea producing) |
| superpathway of 4-aminobutyrate degradation |
| L-alanine degradation I |
| L-glutamine degradation II |
| L-isoleucine degradation I |
| L-leucine degradation I |
| L-phenylalanine degradation V |
| L-tyrosine degradation IV (to 4-methylphenol) |
| methanol oxidation to formaldehyde II |
| λ-carrageenan degradation |
| 2-O-α-mannosyl-D-glycerate degradation |
| cellulose and hemicellulose degradation (cellulolosome) |
| glucose and glucose-1-phosphate degradation |
| ribose degradation |
| trehalose degradation II (trehalase) |
| xylose degradation I |
| heme degradation |
| ammonia assimilation cycle III |
| sulfate reduction I (assimilatory) |
| adenosine nucleotides degradation II |
| pseudouridine degradation |
| seed germination protein turnover |
| wound-induced proteolysis I |
| baicalein degradation (hydrogen peroxide detoxification) |
| furcatin degradation |
| linustatin bioactivation |
| luteolin triglucuronide degradation |
| mannitol degradation I |
| neolinustatin bioactivation |
| phytate degradation I |
| phytate degradation II |
| vicianin bioactivation |
| xylitol degradation |
| triclosan resistance |
| aerobic respiration II (cytochrome c) (yeast) |
| glycolysis I (from glucose 6-phosphate) |
| glycolysis VI (metazoan) |
| C4 photosynthetic carbon assimilation cycle, NAD-ME type |
| C4 photosynthetic carbon assimilation cycle, NADP-ME type |
| C4 photosynthetic carbon assimilation cycle, PEPCK type |
| 2-oxoisovalerate decarboxylation to isobutanoyl-CoA |
| superpathway of glyoxylate bypass and TCA |
| TCA cycle I (prokaryotic) |
| mannosyl-glycoprotein N-acetylglucosaminyltransferases |
| terminal O-glucans residues modification |
| sporopollenin precursor biosynthesis |
| superpathway of 1D-myo-inositol hexakisphosphate biosynthesis (plants) |
| superpathway of phosphatidate biosynthesis (yeast) |
| superpathway of sulfate assimilation and cysteine biosynthesis |
| L-ornithine biosynthesis |
| hesperitin glycoside biosynthesis |
| pinobanksin biosynthesis |
| naringenin glycoside biosynthesis |
