# Supplementary material for: SolCyc: a database hub at the Sol Genomics Network (SGN) for the manual curation of metabolic networks in Solanum and Nicotiana specific databases
Source: Database (Oxford). 2018 May 10;2018:bay035. doi: 10.1093/database/bay035 (PMC5946812; doi:10.1093/database/bay035)
Supplement: Supplementary Data [file bay035_supp.zip › tab 1 S.pdf]

Database	Version	Taxonomic range	# of base pathways	# of superpathways	% of pathways with experimental evidence	source
MetaCyc	20.1	All domains of life	2492	380	96.8	MetaCyc
SolanaCyc	1.0	Solanaceae	199	29	89.0	SGN
PlantCyc	11.0	*Viridiplantae*	1094	119	79.4	PMN
NicotianaCyc	1.0	*Nicotiana*	72	13	74.1	SGN
AraCyc	14.0	*Arabidopsis thaliana*	542	77	62.8	PMN
SoyCyc	7.0	*Glycine max*	531	65	18.1	PMN
CornCyc	7.0	*Zea mays*	484	61	15.4	PMN
NtabacumCyc	1.0	*Nicotiana tabacum*	446	58	14.3	SGN
ChlamyCyc	6.0	*Chlamydomonas reinhardtii*	329	44	12.6	PMN
PoplarCyc	9.0	*Populus trichocarpa*	535	64	8.5	PMN
MaizeCyc	2.2.19	*Zea mays*	424	55	8.1	Gramene
RiceCyc	3.3.1.2.19	*Oryza sativa*	308	50	6.7	Gramene
GrapeCyc	6.0	*Vitis vinifera*	495	62	5.7	PMN
OryzaCyc	4.0	*Oryza sativa*	483	60	5.2	PMN
PapayaCyc	5.0	*Carica papaya*	475	63	3.3	PMN
CassavaCyc	6.0	*Manihot esculenta*	503	64	2.1	PMN
TomatoCyc	2.0	*Solanum lycopersicum*	518	70	2.0	PMN
MossCyc	5.0	*Physcomitrella patens*	473	62	1.3	PMN
PotatoCyc	3.0	*Solanum tuberosum*	493	65	1.3	PMN
SorghumBicolorCyc	4.0	*Sorghum bicolor*	477	63	1.1	PMN
BarleyCyc	4.0	*Hordeum vulgare*	478	62	0.9	PMN
ChineseCabbageCyc	4.0	*Brassica napa ssp. pekinensis*	471	62	0.4	PMN
BrachypodiumCyc	4.0	*Brachypodium distachyon*	470	61	0.2	PMN
SetariaCyc	4.0	*Setaria italic*	482	67	0.2	PMN
SwitchgrassCyc	4.0	*Panicum virgatum*	521	66	0.2	PMN
MedicCyc	16.0	*Medicago truncatula*	374	33	?	Noble
WheatACyc	2.0	*Triticum urartu*	487	64	0	PMN
WheatDCyc	2.0	*Aegilops tauschii*	479	61	0	PMN
SpirodelaCyc	2.0	*Spirodela polyrhiza*	437	59	0	PMN
SelaginellaCyc	5.0	*Selaginella moellendorffii*	416	65	0	PMN
LycoCyc	3.3	*Solanum lycopersicum*	456	63	0	SGN
PotatoCyc	2.1	*Solanum tuberosum*	419	58	0	SGN
PetCyc	2.4	*Petunia x hybrida*	130	54	0	SGN
BenthamianaCyc	2.0	*Nicotiana benthamiana*	344	41	0	SGN
CoffeaCyc	2.4	*Coffea sp.*	312	39	0	SGN
