# Supplementary material for: SolCyc: a database hub at the Sol Genomics Network (SGN) for the manual curation of metabolic networks in Solanum and Nicotiana specific databases
Source: Database (Oxford). 2018 May 10;2018:bay035. doi: 10.1093/database/bay035 (PMC5946812; doi:10.1093/database/bay035)
Supplement: Supplementary Data [file bay035_supp.zip › table 2 S.pdf]

glycine betaine biosynthesis I (Gram-negative bacteria)
glycine betaine biosynthesis V (from glycine)
histamine biosynthesis
diphthamide biosynthesis (archaea)
L-citrulline biosynthesis
protein citrullination
<i>S</i>-adenosyl-L-methionine cycle I
glycine biosynthesis II
glycine biosynthesis IV
L-asparagine biosynthesis III (tRNA-dependent)
L-citrulline-nitric oxide cycle
L-glutamate biosynthesis III
L-phenylalanine biosynthesis I
L-proline biosynthesis I
L-selenocysteine biosynthesis II (archaea and eukaryotes)
L-tyrosine biosynthesis IV
2,3-dihydroxybenzoate biosynthesis
acetaldehyde biosynthesis I
CMP-3-deoxy-D-manno-octulosonate biosynthesis II (from D-arabinose 5-phosphate)
GDP-L-fucose biosynthesis II (from L-fucose)
glycogen degradation I
superpathway of UDP-glucose-derived O-antigen building blocks biosynthesis
UDP-α-D-glucuronate biosynthesis (from myo-inositol)
UDP-L-arabinose biosynthesis II (from L-arabinose)
peptidoglycan biosynthesis I (meso-diaminopimelate containing)
peptidoglycan biosynthesis III (mycobacteria)

coenzyme A biosynthesis II (mammalian)
lipoate salvage I
chlorophyllide a biosynthesis III (aerobic, light independent)
1,4-dihydroxy-2-naphthoate biosynthesis II (plants)
menaquinol-6 biosynthesis
menaquinol-8 biosynthesis
menaquinol-9 biosynthesis
γ-glutamyl cycle
L-ascorbate biosynthesis V
arachidonate biosynthesis IV (8-detaturase)
cyclopropane fatty acid (CFA) biosynthesis
docosahexaenoate biosynthesis III (mammals)
fatty acids biosynthesis (yeast)
icosapentaenoate biosynthesis V (Δ8 desaturase)
linoleate biosynthesis II (animals)
palmitoleate biosynthesis I (*E. coli*)
palmitoleate biosynthesis III (cyanobacteria)
stearate biosynthesis III (fungi)
3-phosphoinositide biosynthesis
phosphatidate metabolism, as a signaling molecule
phosphatidylcholine biosynthesis V
phosphatidylserine biosynthesis II
cholesterol biosynthesis I
cholesterol biosynthesis II (via 24,25-dihydrolanosterol)
cholesterol biosynthesis III (via desmosterol)
lanosterol biosynthesis
superpathway of cholesterol biosynthesis
monoacylglycero metabolism (yeast)
ethylene biosynthesis III (microbes)

serotonin and melatonin biosynthesis
pyrimidine deoxyribonucleotides *de novo* biosynthesis III
mRNA capping I
tRNA methylation (yeast)
tRNA splicing
adenine and adenosine salvage IV
pyrimidine deoxyribonucleotides biosynthesis from CTP
eumelanin biosynthesis
L-dopachrome biosynthesis
autoinducer AI-1 biosynthesis
dehydroscoulerine biosynthesis
dhurrin biosynthesis
papaverine biosynthesis
sanguinarine and macarpine biosynthesis
apigeninidin 5-O-glucoside biosynthesis
epoxypseudoisoeugenol-2-methylbutyrate biosynthesis
flavonoid biosynthesis (in equisetum)
gentiodelphin biosynthesis
justicidin B biosynthesis
luteolinidin 5-O-glucoside biosynthesis
medicarpin biosynthesis
phenylpropanoids methylation (ice plant)
pterostilbene biosynthesis
quercetin sulfate biosynthesis
rose anthocyanin biosynthesis II (via cyanidin 3-O-β-D-glucoside)
rosmarinic acid biosynthesis II
salvianin biosynthesis
superpathway of anthocyanin biosynthesis (from delphinidin 3-O-glucoside)

superpathway of anthocyanin biosynthesis (from pelargonidin 3-O-glucoside)
ternatin C5 biosynthesis
kauralexin biosynthesis
oryzalide A biosynthesis
1D-myo-inositol hexakisphosphate biosynthesis II (mammalian)
D-myo-inositol (1,3,4)-trisphosphate biosynthesis
inositol pyrophosphates biosynthesis
costunolide biosynthesis
linalool biosynthesis II
soybean saponin I biosynthesis
superpathway avenacin A biosynthesis
valencene and 7-epi-α-selinene biosynthesis
hypoglycin biosynthesis
salidroside biosynthesis
2'-deoxymugineic acid phytosiderophore biosynthesis
enterobactin biosynthesis
glycerol degradation III
methylglyoxal degradation II
4-aminobutyrate degradation II
4-aminobutyrate degradation III
allantoin degradation to ureidoglycolate I (urea producing)
superpathway of 4-aminobutyrate degradation
L-alanine degradation I
L-glutamine degradation II
L-isoleucine degradation I
L-leucine degradation I
L-phenylalanine degradation V

L-tyrosine degradation IV (to 4-methylphenol)
methanol oxidation to formaldehyde II
λ-carrageenan degradation
2-O-α-mannosyl-D-glycerate degradation
cellulose and hemicellulose degradation (cellulolosome)
glucose and glucose-1-phosphate degradation
ribose degradation
trehalose degradation II (trehalase)
xylose degradation I
heme degradation
ammonia assimilation cycle III
sulfate reduction I (assimilatory)
adenosine nucleotides degradation II
pseudouridine degradation
seed germination protein turnover
wound-induced proteolysis I
baicalein degradation (hydrogen peroxide detoxification)
furcatin degradation
linustatin bioactivation
luteolin triglucuronide degradation
mannitol degradation I
neolinustatin bioactivation
phytate degradation I
phytate degradation II
vicianin bioactivation
xylitol degradation
triclosan resistance
aerobic respiration II (cytochrome c) (yeast)
glycolysis I (from glucose 6-phosphate)
glycolysis VI (metazoan)

C4 photosynthetic carbon assimilation cycle, NAD-ME type
C4 photosynthetic carbon assimilation cycle, NADP-ME type
C4 photosynthetic carbon assimilation cycle, PEPCK type
2-oxoisovalerate decarboxylation to isobutanoyl-CoA
superpathway of glyoxylate bypass and TCA
TCA cycle I (prokaryotic)
mannosyl-glycoprotein N-acetylglucosaminyltransferases
terminal O-glucans residues modification
sporopollenin precursor biosynthesis
superpathway of 1D-myo-inositol hexakisphosphate biosynthesis (plants)
superpathway of phosphatidate biosynthesis (yeast)
superpathway of sulfate assimilation and cysteine biosynthesis
L-ornithine biosynthesis
hesperitin glycoside biosynthesis
pinobanksin biosynthesis
naringenin glycoside biosynthesis
