# Supplementary material for: SolCyc: a database hub at the Sol Genomics Network (SGN) for the manual curation of metabolic networks in Solanum and Nicotiana specific databases
Source: Database (Oxford). 2018 May 10;2018:bay035. doi: 10.1093/database/bay035 (PMC5946812; doi:10.1093/database/bay035)
Supplement: Supplementary Data [file bay035_supp.zip › legends to supplemental figures and tables.docx]

Legends Supplemental Figures and Tables

Supplemental Figure 1A MetaCyc pathway breakdown into metabolic categories

Supplemental Figure 1B PlantCyc pathway breakdown into metabolic categories

Supplemental Figure 2 Pathway network Venn diagram. Cross-over of predicted pathways between *Nicotiana tabacum*, *Nicotiana sylvestris* and *Nicotiana tomentosiformis*. Number and percentage for shared and unique pathways are shown in each intersection. **The pathway numbers refer to all pathways of the species including superpathways.**

Supplemental Figure 3 Numbers and metabolic mapping of *Nicotiana tabacum* experimentally validated pathways in SolanaCyc. The numbers in parentheses are the overall counts for the metabolic categories in SolanaCyc.

Supplemental Tables

Supplemental Table 1 Overview of selected MetaCyc-derived plant databases

Supplemental Table 2 List of predicted pathways in NtabacumCyc, which have been curated invalid for the species **(131 base pathways and 25 superpathways)**
