# Supplementary material for: SolCyc: a database hub at the Sol Genomics Network (SGN) for the manual curation of metabolic networks in Solanum and Nicotiana specific databases
Source: Database (Oxford). 2018 May 10;2018:bay035. doi: 10.1093/database/bay035 (PMC5946812; doi:10.1093/database/bay035)
Supplement: Supplementary Data [file bay035_supp.zip › supplemental figures.docx]

Supplemental Figures


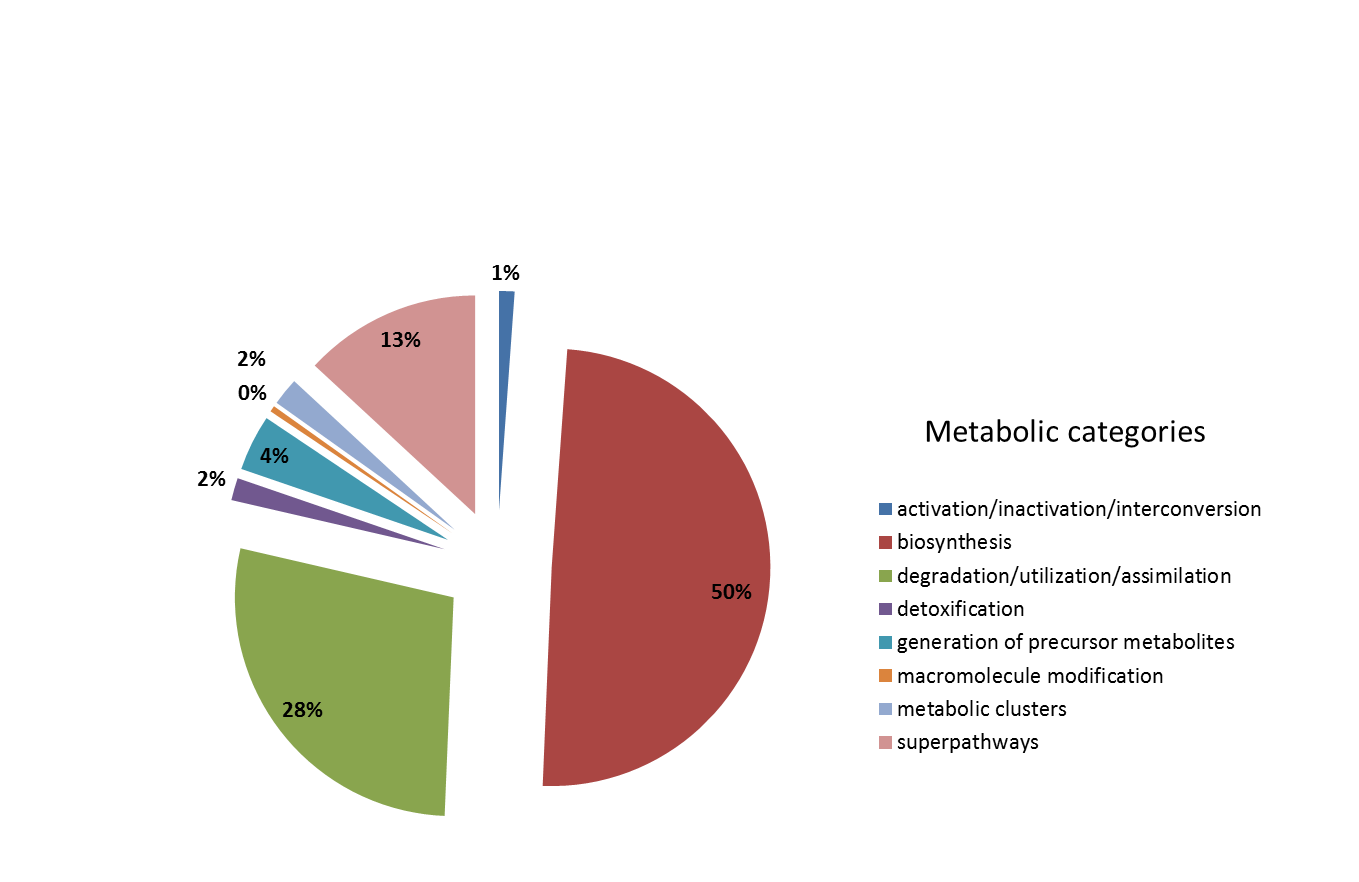


Supplemental Figure 1A MetaCyc pathway breakdown into metabolic categories


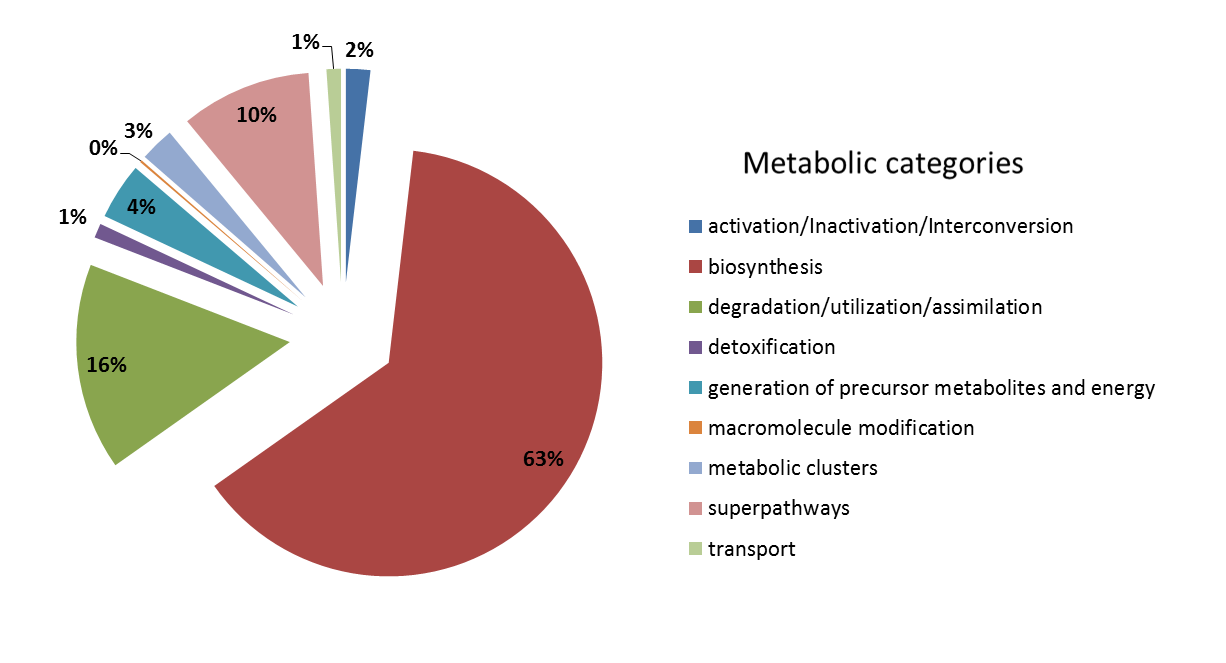
Supplemental Figure 1B PlantCyc pathway breakdown into metabolic categories


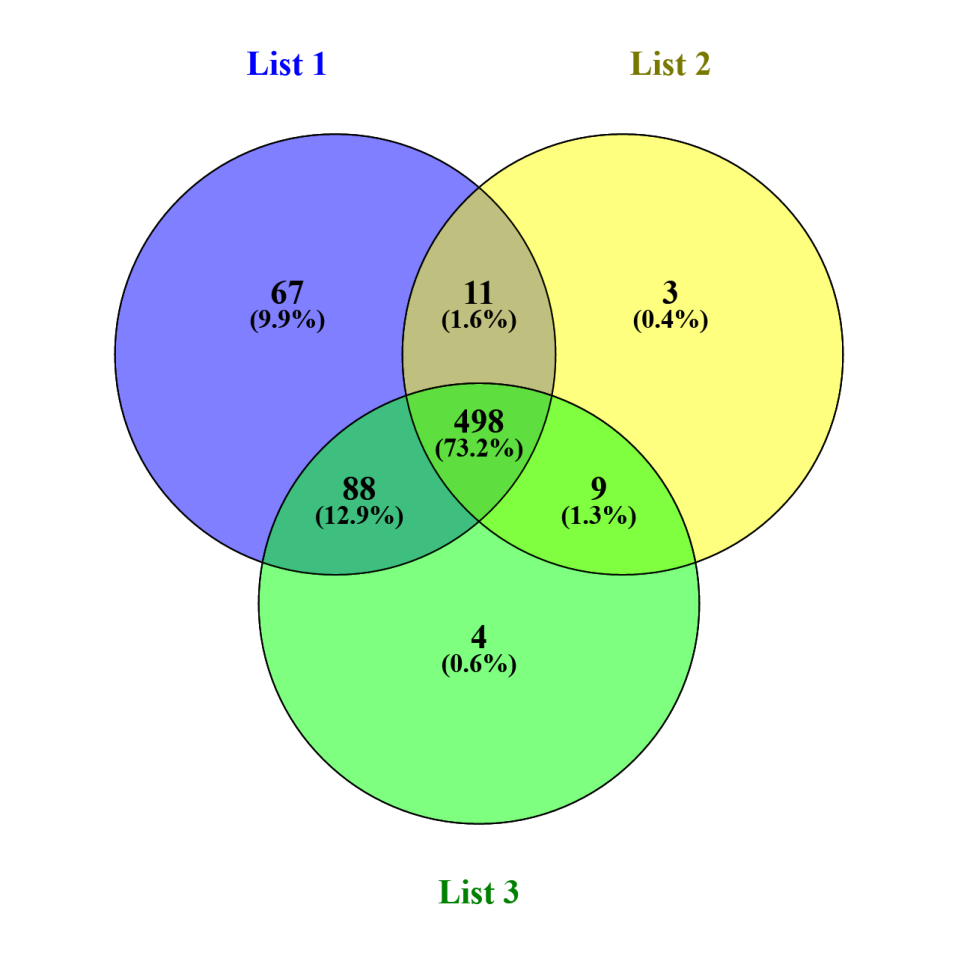


*Nicotiana tabacum*

*Nicotiana sylvestris*

*Nicotiana tomentosiformis*

Supplemental Fig. 2 Pathway network VENN diagram. Cross-over of predicted pathways between *Nicotiana tabacum*, *Nicotiana sylvestris* and *Nicotiana tomentosiformis*. Number and percentage for shared and unique pathways are shown in each intersection.


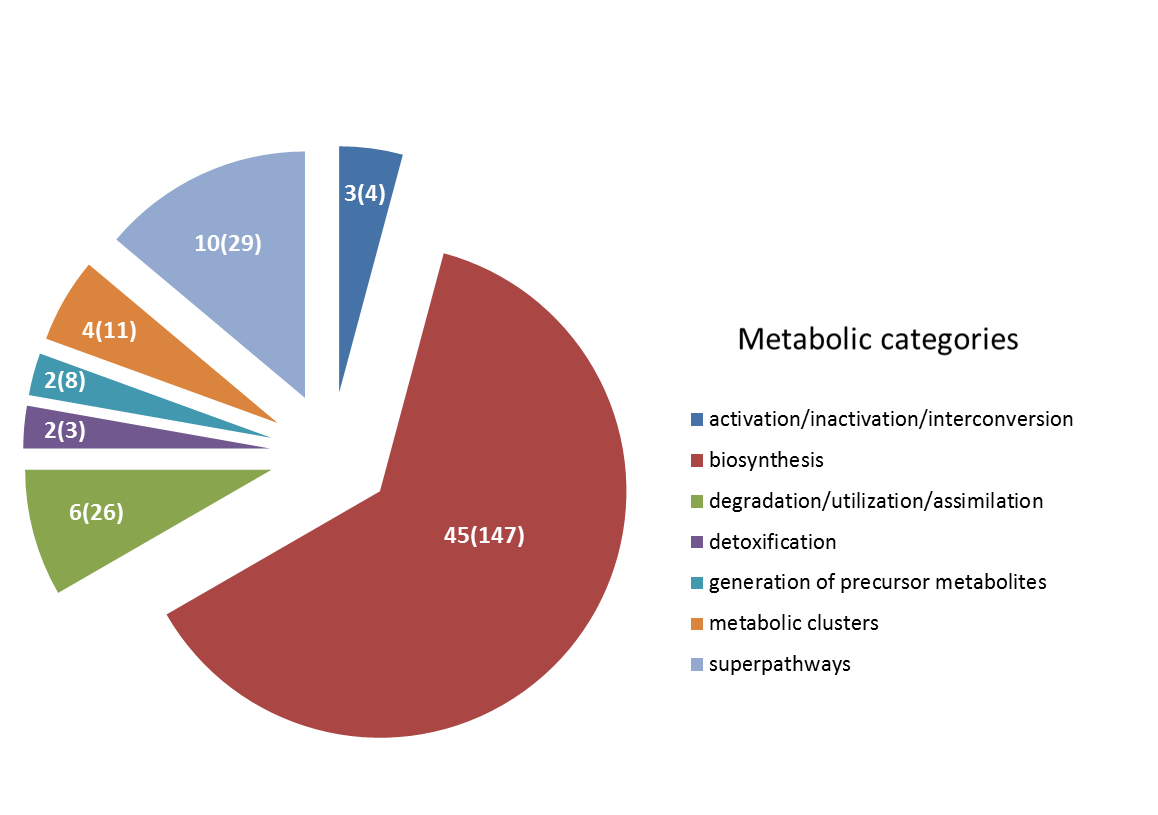


Supplemental Fig. 3 Numbers and metabolic mapping of *Nicotiana tabacum* experimentally validated pathways in SolanaCyc. The numbers in parentheses are the overall counts for the metabolic categories in SolanaCyc.
