# Supplementary figures and images for: SolCyc: a database hub at the Sol Genomics Network (SGN) for the manual curation of metabolic networks in Solanum and Nicotiana specific databases
Source: Database (Oxford). 2018 May 10;2018:bay035. doi: 10.1093/database/bay035 (PMC5946812; doi:10.1093/database/bay035)

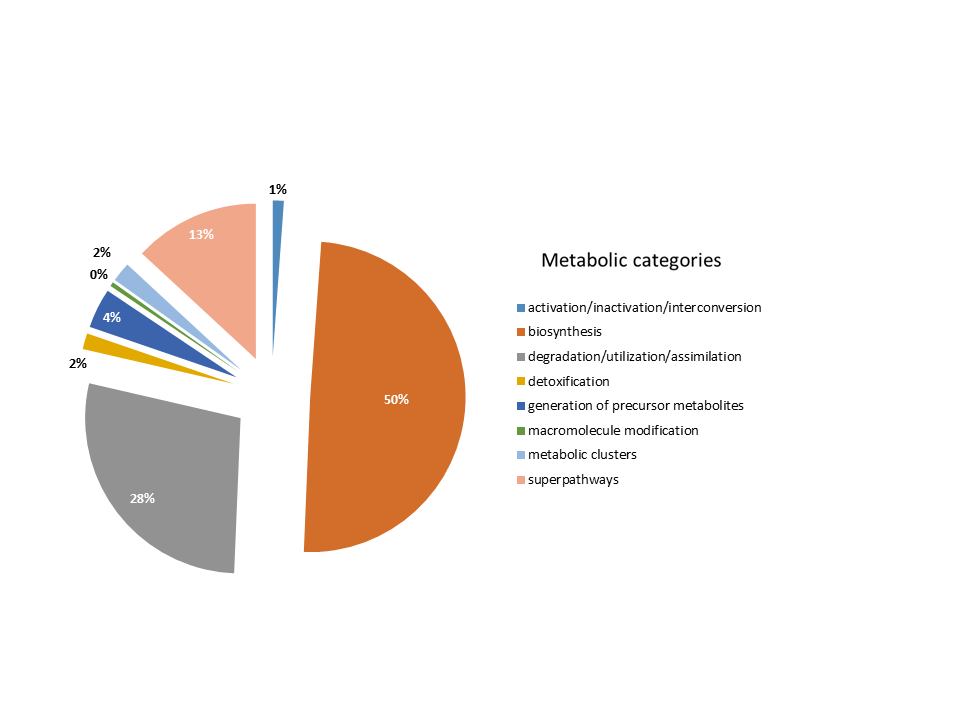

Supplement: Supplementary Data [file bay035_supp.zip › Fig 1A S.tif]

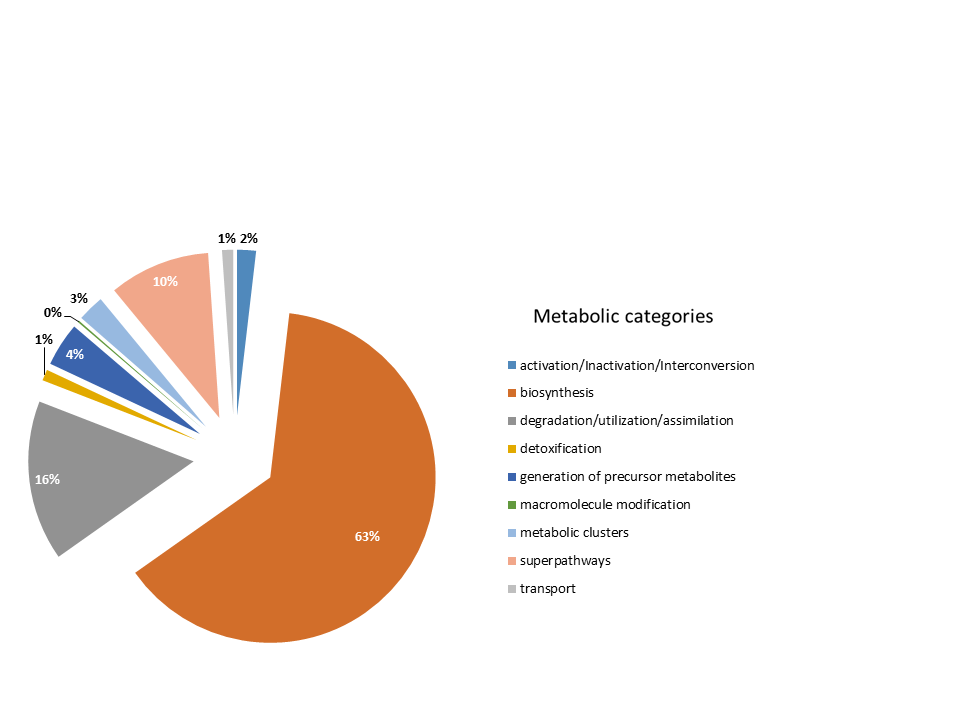

Supplement: Supplementary Data [file bay035_supp.zip › Fig 1B S.tif]

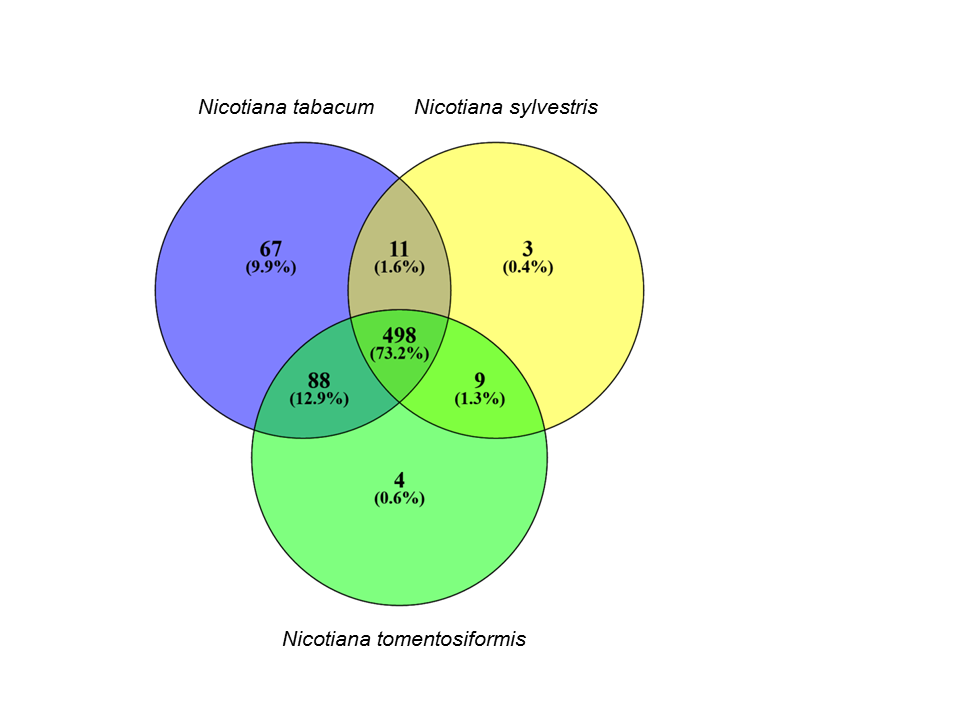

Supplement: Supplementary Data [file bay035_supp.zip › Fig 2 S.tif]

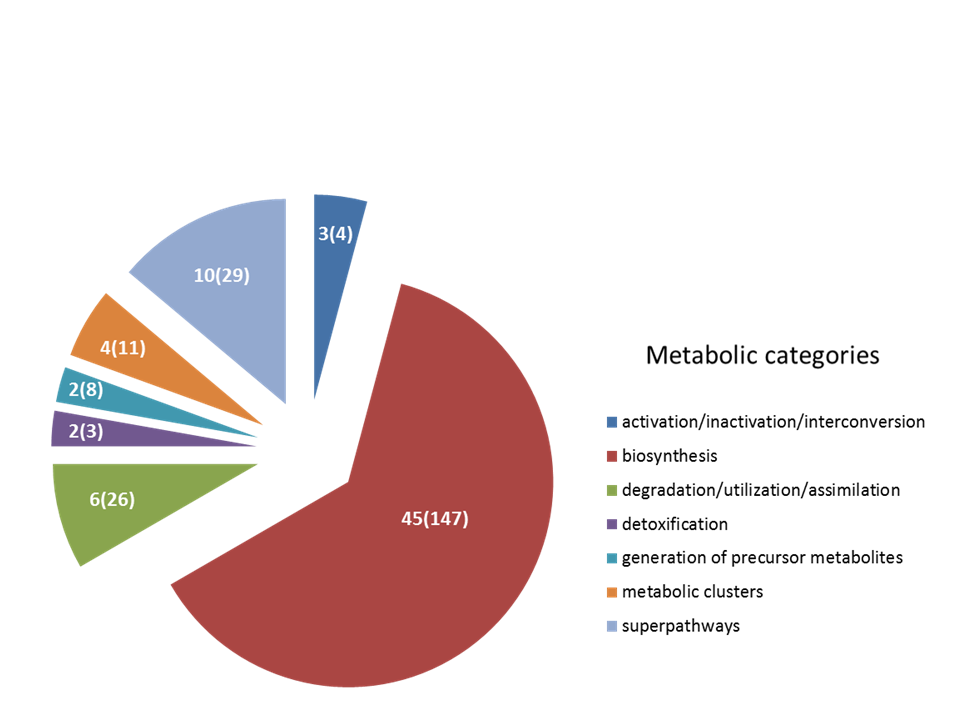

Supplement: Supplementary Data [file bay035_supp.zip › Fig 3 S.tif]
